# Canopy bird assemblages are less influenced by habitat age and isolation than understory bird assemblages in Neotropical secondary forest

**DOI:** 10.1002/ece3.4086

**Published:** 2018-04-27

**Authors:** Tom Bradfer‐Lawrence, Nick Gardner, Daisy H. Dent

**Affiliations:** ^1^ School of Biological and Environmental Sciences University of Stirling Stirling UK; ^2^ Smithsonian Tropical Research Institute Panama City Panama

**Keywords:** avian, colonization, conservation, Panama, reassembly, secondary forest

## Abstract

Secondary forest habitats are increasingly recognized for their potential to conserve biodiversity in the tropics. However, the development of faunal assemblages in secondary forest systems varies according to habitat quality and species‐specific traits. In this study, we predicted that the recovery of bird assemblages is dependent on secondary forest age and level of isolation, the forest stratum examined, and the species’ traits of feeding guild and body mass. This study was undertaken in secondary forests in central Panama; spanning a chronosequence of 60‐, 90‐, and 120‐year‐old forests, and in neighboring old‐growth forest. To give equal attention to all forest strata, we employed a novel method that paired simultaneous surveys in canopy and understory. This survey method provides a more nuanced picture than ground‐based studies, which are biased toward understory assemblages. Bird reassembly varied according to both habitat age and isolation, although it was challenging to separate these effects, as the older sites were also more isolated than the younger sites. In combination, habitat age and isolation impacted understory birds more than canopy‐dwelling birds. Proportions of dietary guilds did not vary with habitat age, but were significantly different between strata. Body mass distributions were similar across forest ages for small‐bodied birds, but older forest supported more large‐bodied birds, probably due to control of poaching at these sites. Canopy assemblages were characterized by higher species richness, and greater variation in both dietary breadth and body mass, relative to understory assemblages. The results highlight that secondary forests may offer critical refugia for many bird species, particularly specialist canopy‐dwellers. However, understory bird species may be less able to adapt to novel and isolated habitats and should be the focus of conservation efforts encouraging bird colonization of secondary forests.

## 1 INTRODUCTION

Eight million hectares of old‐growth tropical forest were degraded or destroyed each year between 1990 and 2015 (FAO, 2015). Yet currently there is a pantropical expansion in the area of secondary forest, as pastureland is abandoned and selectively logged forest recovers (Aide et al., 2013; FAO, 2015; Wright, 2005; Wright & Muller‐Landau, 2006). These regenerating secondary forest habitats may have the potential to act as refugia for tropical forest species, mitigating the loss of old‐growth forest (Dent & Wright, 2009; Koh & Gardner, 2010). Therefore, accurately quantifying the conservation value of secondary forest is crucial, both to inform environmental policy at national and international levels and to improve and direct conservation actions such as habitat restoration for threatened fauna (Anderson, 2009; Chazdon et al., 2009; Dunn, 2004).

Many studies examining the conservation potential of secondary forest have focused on birds (Borges, 2007; Petit, Petit, Christian, & Powell, 1999; Powell, Stouffer, & Johnson, 2013; Sberze, Cohn‐Haft, & Ferraz, 2010), as they are relatively easy to study, their taxonomy is well documented, and they respond quickly to changing environmental conditions (Gregory et al., 2005; Vanderwalle et al., 2010). The conservation value of secondary forest can be inferred from the presence of bird species dependent on features typical of old‐growth forest, such as large trees or epiphytes (Dent, 2010; Gardner et al., 2008; Lees & Peres, 2008, 2010). Moreover, as birds are important contributors to ecosystem services such as seed dispersal, the presence of key forest specialists can be a useful indicator of future habitat development (Pejchar et al., 2008; Winkler & Preleuthner, 2001; Wunderle, 1997). These studies have demonstrated that secondary habitats can host a high proportion of tropical forest fauna, and therefore these habitats may play a vital role in the conservation of old‐growth species (Dent, 2010).

Despite secondary forests supporting many old‐growth species, it is still unclear when, or even if, secondary forest habitats can become functionally analogous with old‐growth ecosystems (Dent, 2010; Guariguata & Ostertag, 2001). Some avian groups, such as understory insectivores, have been the focus of research regarding rates of species colonization and reassembly (Powell et al., 2013, 2015; Stouffer & Bierregaard, 1995, 2007). Many understory species are strongly dispersal limited and are incapable of crossing even small gaps in forest cover (Laurance, 2004; Laurance, Stouffer, & Laurance, 2004; Lees & Peres, 2009; Moore, Robinson, Lovette, & Robinson, 2008; Stratford & Stouffer, 1999). However, Neotropical birds are an enormously diverse group, so secondary forest colonization rates are likely to vary greatly among different bird species and between the assemblages in different forest strata (Laurance et al., 2004). In contrast to understory species, many canopy species are able to disperse across open areas (Burney & Brumfield, 2009; Graham, 2001; Graham & Blake, 2001; Peters & Nibbelink, 2011) and hence reassembly might be faster in the canopy compared to the understory in isolated forests. Alternatively, reassembly in the canopy may be limited because many canopy species require an advanced level of structural complexity or resource availability before they can inhabit an area (Nadkarni, 1994; Nadkarni & Matelson, 1989).

Similarly, species’ colonization of secondary forest will be mediated by traits such as dispersal ability and dietary niche (Newbold et al., 2013). Larger birds can generally fly further than smaller species, for example, toucans and parrots have large ranges and will readily commute between different forest patches when foraging (Graham, 2001; Willis & Eisenmann, 1979). Likewise, generalist consumers are more likely to find suitable foraging in younger forest, compared with those species with narrower dietary niches, which might struggle to establish if their food sources are not yet present (Boyle & Sigel, 2015; Powell et al., 2013, 2015; Stouffer, Johnson, Bierregaard, & Lovejoy, 2011). Improved knowledge of the subtleties underlying development of a bird assemblage in the different dietary guilds and forest strata at a single site will enhance understanding of secondary forest systems and facilitate maximization of the conservation value of secondary habitats, revealing where and when interventions are required.

Acquiring such fine‐scale knowledge of reassembly dynamics in these habitats is challenging because bird research is conducted almost exclusively on the ground, while tropical forests are structurally complex with high canopies. Many bird species live or forage in the canopy (Nadkarni & Matelson, 1989; Pearson, 1971; Winkler & Preleuthner, 2001) and most survey methods tend to underestimate the presence and abundance of species found in upper levels of the forest (Anderson, 2009; Blake, 2007; Walther, 2003; Winkler & Preleuthner, 2001). In consequence using solely ground‐based survey methods means that much of the complexity of bird assemblages remains unrecorded (Walther, 2003). While there is a reasonable understanding of some of the factors influencing the persistence of specific groups of understory bird species in degraded tropical forests (Visco et al., 2015), there is almost no comparable research for canopy species.

Of the limited number of studies explicitly examining bird assemblages in tropical forest canopies, most have been located solely in old‐growth forest (Anderson, 2009; Loiselle, 1988; Naka, 2004), and the one canopy study conducted in secondary forest presents no comparison with old‐growth habitat (Greenberg, 1981). Critically, canopy‐based studies have, thus far, concentrated exclusively on the birds of the canopy, mirroring the issues associated with understory‐focused studies. No study has considered how secondary forest bird assemblages might vary spatially or temporally within discrete forest strata and hence cannot explain the differences between the canopy and understory bird assemblages that exist within a single forest stand. Without an accurate assessment of the complete bird assemblage—understory and canopy—the explanatory power of any study is restricted to the stratum of focus. Moreover, no research has investigated the impact that the different environmental conditions in each habitat strata have on the colonization and persistence of different bird species in secondary forest. Some studies have hypothesized that forest age and extent of disturbance might underlie compositional dissimilarities in canopy bird assemblages observed at different sites in the Neotropics (Loiselle, 1988; Naka, 2004), but to date, there has been no explicit assessment of this.

Here, we employed a novel, paired canopy and understory survey methodology to examine bird reassembly across a chronosequence of regenerating secondary forest in central Panama. We addressed the following specific hypotheses, that avian colonization of secondary forests would (1) be dictated by both forest age and level of stand isolation, with more advanced stages of reassembly in older and more connected sites; (2) vary between strata, with canopy assemblages showing more advanced stages of reassembly in younger sites than understory assemblages; and (3) vary according to the species‐specific traits of feeding guild and body mass, where larger species with generalist diets will colonize more rapidly than smaller species with specialist diets.

## 2 METHODS

### 2.1 Study site

This study was conducted in the Barro Colorado Nature Monument (BCNM) in central Panama (Figure S1). The BCNM includes tracts of old‐growth forest and areas of secondary forest with a range of ages and a well‐documented history of past land use (Denslow & Guzman, 2000; Piperno, 1990). We surveyed birds in four forest age classes: 60‐, 90‐, 120‐year‐old secondary forest, and old‐growth forest, with sites identified from maps and aerial photographs. The old‐growth forests on Barro Colorado Island are thought to be at least 500 years old, and there is no evidence that these forests have ever been logged or cultivated (Piperno, 1990). The secondary forests were used for cattle farming or fruit production prior to land abandonment (Dent, DeWalt, & Denslow, 2013). In the secondary forests, canopy height and number of large trees increase with habitat age, whereas density of understory vegetation declines. Further details of vegetation structure and composition can be found in Dent et al. (2013), DeWalt, Maliakal, and Denslow (2003) and Mascaro, Asner, Dent, DeWalt, and Denslow (2012). Sites were selected to span a range in forest age and isolation (island and mainland; see Figure S1 and Table S1). Full replication of forest age and isolation was not possible due to the land‐use history of the region, and all of the older sites were on BCI, while the younger sites were on neighboring peninsulas.

Twelve trees were selected to act as focal survey points, three in each age class. No forest younger than 60 years old was included as there were no trees suitable to act as the focal survey points. Focal survey trees were identified in each forest stand; all were either *Dipteryx panamensis* or *Pseudobombax septenatum* as these are suitably safe for climbing. Trees were at least 150 m from the forest patch edge, and a minimum of 400 m from other survey trees to ensure that sites were independent as far as possible. Given the large home ranges of some bird species (Robinson, Brawn, & Robinson, 2000), there is a possibility that the same individuals were detected from more than one tree but, considering repeat visits were made to each tree, it is unlikely that this led to systematic inflation of population estimates. The canopy observer used the single rope technique to climb trees (Anderson, Koomjian, French, Altenhoff, & Luce, 2015; Coffey & Andersen, 2012). Prior to their inclusion as focal survey points, all trees were assessed during daylight hours for their safety and suitability, particularly to ensure that they had an open crown structure offering a good view over the surrounding forest (Anderson, 2009).

### 2.2 Data collection

Data were collected during the dry season between February and April 2015, when Neotropical migrants were present. Due to the time required to enter the canopy, data collection was limited to a single site per day. Therefore, this study adopted a method used in previous canopy bird research, whereby a single, continuous survey is subdivided into shorter time blocks (Anderson, 2009; Naka, 2004). However, in a significant variation from the methodology used in these studies, there were two observers at every count, one in the canopy and one on the ground.

Each of the 12 sites was visited 5 times over the 3‐month study period, giving a total of 60 surveys. Both the canopy and ground observers followed the same protocol. During each survey all birds seen or heard up to 150 m away were recorded (total area 7.1 ha: Anderson, 2009; Naka, 2004). Observers did not record raptors, hirundines or swifts flying overhead, nor those species associated with the nearby aquatic habitats of Lake Gatun (Angehr & Dean, 2010). Surveys commenced at nautical twilight; approximately 45 min before sunrise (times taken from https://www.timeanddate.com). Each survey lasted for three hours, which was split into 36 consecutive 5‐min blocks. The use of these short time blocks facilitated tracking of individual birds, reducing the possibility of double counting (Anderson, 2009; Loiselle, 1988; Naka, 2004). Data from both observers were combined following each survey. The species count for one survey was the maximum number of individuals detected in any single 5‐min time‐block by either one of the observers. The species count data from each survey was then summed to give the total number of individuals per species per site over all 5 surveys.

The first instance an individual bird was detected during a survey the following data were recorded: species identity; method of detection (aural only, visual only, or aural and visual); horizontal distance from observer (bands 0–5, 5–10, 10–25, 25–50, 50–100 m, and >100 m, confirmed with a laser rangefinder where possible); and forest stratum. The forest was divided into three strata: the understory (Ground to 3 m), mid‐level (from 3 m to below the canopy), and canopy (the top layer of vegetation and any emergent crowns; adapted from Anderson, 2009). If the bird was only heard, then the observers were required to estimate the stratum position. Based on assignments from the entire dataset, species were later categorized as inhabiting one of the three strata by employing the utilization‐availability analysis method of Neu, Byers, and Peek (1974). Briefly, chi‐squared goodness‐of‐fit tests with Bonferroni corrections of the significance level were used to determine whether the observed number of detections was greater than expected in a particular stratum for each species (Anderson, 2009). A significant preference for a particular stratum was indicated by expected values below the 95% confidence limits for the observed values (Anderson & Naka, 2011; Cardoso da Silva, Uhl, & Murray, 1996). Any species with fewer than three detections, or which did not show a significant difference between expected and observed detections was assigned to the mid‐level assemblage. This ensured that the species assigned to the understory or canopy assemblages were “core” members (*sensu* Cohn‐Haft, Whittaker, & Stouffer, 1997; Naka, 2004; Anderson & Naka, 2011) that spend the majority of their time in those strata, whereas species with no clear preference were assigned to the mid‐level assemblage. There was a high level of concordance between assignments when using aural compared to visual detections, and between those based on the dataset and details in the published literature (Angehr & Dean, 2010; Ridgely & Gwynne, 1989).

All species were assigned to one of six broad feeding guilds used by Anderson and Naka (2011): frugivore, granivore, insectivore, omnivore, nectarivore, and raptor. Assignment was based on dietary information in Angehr and Dean (2010), and Ridgely and Gwynne (1989). Body mass estimates were collated from del Hoyo, Elliott, Sargatal, Christie, and de Juana (2015). Dependence on forest habitats was assigned according to details in Stotz, Fitzpatrick, Parker, and Moskovitz (1996). Categorization was highly conservative; only those species with “F1—Tropical Lowland Evergreen Forest” listed as their primary preferred habitat were classed as forest specialists. A species list with these details can be found in Table S2.

Audio recordings of the complete survey were made by both observers to facilitate subsequent identification of any unknown bird calls (using a Zoom H4N digital recorder and Sennheiser ME66 microphone). Surveys were only undertaken when there was no rain, and when the wind in the canopy was below 2.5 m/s (assessed using a handheld anemometer; Proster, model TL017). While bird activity is frequently still high under these environmental conditions, the observers were unable to survey accurately due to the increased background noise. Although the two observers (TBL and NG) did not change roles between canopy and understory during the study, every effort was made to ensure comparable levels of knowledge and detection ability. Both observers have considerable ornithological field experience (over 10 years each), including in tropical forest habitats. Prior to the study, both observers spent 6 weeks familiarizing themselves with local bird calls. Once in the field, over 100 hours of formal and informal training took place before data collection began, including detection tests to check for any bias in aural identification ability and for consistency and accuracy in estimations of distance. Further practice then continued throughout the field season to guarantee maintenance of equivalent skills and knowledge.

### 2.3 Data analyses

Rarefaction curves were used to compare rates of species accumulation among forest age classes, and assess survey completeness. Distance sampling was used to determine whether there were comparable levels of detection among sites (Buckland, Rexstad, Marques, & Oedekoven, 2015; Marques, Thomas, Fancy, & Buckland, 2007). Using the complete dataset, conventional distance sampling (CDS) and multiple‐covariate distance sampling (MCDS) with “forest age” and “site” as covariates were performed. Model selection was based on Akaike's information criterion (AIC; Akaike, 1973). Models were ranked according to their AIC value, and those with a difference (ΔAIC) of <2 were considered to be equally supported. The final model did not include either site or habitat age, suggesting that was no consistent difference in detectability among sites. Hence, all the subsequent analyses were conducted using the full data set.

All of the following analyses were conducted with abundance data. In this study, we detected twelve species at mainland sites that are known to be absent from BCI due to isolated‐related extirpations (Robinson, 1999, 2001; Willis & Eisenmann, 1979; detailed in Table S2). To disentangle the possible confounding effects of forest age and isolation, some of the analyses were undertaken with these island‐extirpated species removed. These data subsets are referred to as island‐extirpated (IE) datasets hereafter. By contrasting the datasets with and without the island‐extirpated species, some assessment of the impact of isolation on reassembly was possible, particularly in terms of the responses of strata‐specific assemblages.

Nonmetric multi‐dimensional scaling (NMDS) was used to explore patterns in assemblage composition. The NMDS analyses were performed with the abundance‐based Jaccard index. In all cases, the NMDS was implemented with two axes, which conformed to minimum stress requirements (Kruskal, 1964). Analysis was initially undertaken using the complete dataset to study the broader patterns in assemblage composition, with further investigations of the separate canopy and understory assemblages performed using data for the core assemblage members only (as defined above). Using the NMDS dissimilarity matrix, distances between the assemblages were summed within age class to give a measure of dispersion, allowing for quantitative comparison between strata.

Further NMDS were undertaken with the IE datasets to investigate the effect that the extirpations might have on composition of the whole assemblage, and on the understory and canopy assemblages separately. Using the NMDS dissimilarity matrix for the complete dataset, the value for each pairwise distance between sites was subtracted from the equivalent value in the NMDS dissimilarity matrix for the IE dataset. These differences were square‐transformed to make them positive and then summed. To interpret these values, higher values indicate larger distances between assemblages, and thus greater influence of isolation‐related extirpations on composition at any one site. Contrasting the mean distances between the two datasets gave an illustration of the relative impacts that isolation has on the composition of the canopy and understory assemblages at the island sites.

Permutational multivariate analysis of variance (PERMANOVA) was used to investigate the quantitative relationships between assemblage composition and forest age and between assemblage composition and isolation (mainland or island). This was undertaken with the complete dataset and with the canopy and understory assemblages separately. Again, analyses were then repeated with the IE datasets, to identify the impact that isolation might have on assemblage composition.

Generalized linear models (GLM) with binomial errors and a logit link were used to investigate differences in guild proportions between strata and forest age classes at each site. Initial models based on the core understory and canopy assemblages included “guild”, “forest age”, and the “guild” by “forest age” interaction. “forest age” was treated as a categorical variable because of the uncertainty surrounding absolute age of the old‐growth forests, and the relatively small age range of the secondary forest. Further GLMs were performed for each guild individually, with models including “forest age”, “strata”, and the “forest age” by “strata” interaction. Model selection was again based on Akaike's information criterion, corrected for small samples (AICc; Akaike, 1973). The importance of each predictor was assessed by Akaike weight (*w*_*i*_), which indicates the probability that the particular model is the best fit for the data (Burnham & Anderson, 2002).

Body mass patterns were examined by plotting histograms of number of individuals against log‐transformed body mass. Kurtosis and skewness values were calculated for the canopy and understory assemblages separately. Generalized linear models with poisson errors and a log link were used to test for differences in distribution patterns between canopy and understory assemblages and among age classes. Body mass showed a bi‐modal distribution, and so an additional predictor “body size” was also included. This was defined by splitting the data either side of the median mass to give two groups: small‐ and large‐bodied birds. Hence, full models included “forest age”, “strata”, and “body size”. with all possible interactions. Model selection was performed via AIC as described above. This provided an indication of the diversity of the body sizes in the different assemblages, and any bias in their distribution.

All analyses were undertaken using R version 3.2.1 (R Core Team, 2016). The “Distance” package was used for the detectability assessment (Miller, Rexstad, Thomas, Marshall, & Laake, 2016). NMDS and PERMANOVA analyses were performed using the package “vegan” (Oksanen et al., 2016). GLM model simplification was undertaken using the package “MuMIn” (Bartoń, 2016), and kurtosis and skewness were calculated with the package “moments” (Komsta & Novomestky, 2015). Figures were created using the package “ggplot2” (Wickham, 2009).

## 3 RESULTS

We detected 6,223 individuals across 145 species in 34 families (Table S1 and Figure S2). Species accumulation curves suggest that the majority of species had been detected at each site, although some rarer species may have been recorded with further surveys. At the landscape scale, the accumulation curve had reached the asymptote, suggesting that sampling was adequate to accurately describe the community across the study area (Figure S2). Distance sampling model selection indicated that neither forest age nor site explained detectability, suggesting that there was no systematic difference in detectability among sites (Table S3), and thus, all analyses presented here were conducted using the original observations.

Forest specialists accounted for 73%–84% of species, and 72%–84% of individuals, at each site (Table S1). Only eight of the 145 species detected are not generally associated with “F1—Tropical Lowland Evergreen Forest” habitat (Table S2; Stotz et al., 1996). The 90‐year‐old secondary forests had higher species richness than the other forests; species richness in the 60‐, 120‐year‐old, and old‐growth forests were similar (Table S1). This pattern was repeated in the individual strata assemblages. Shannon diversity indices were higher for the 90‐year‐old forests than the other forests, suggesting that the higher species richness in 90‐year‐old forest was driven by a greater number of rare species.

### 3.1 Colonization of secondary forest will vary according to habitat age and isolation

**Figure 1:**
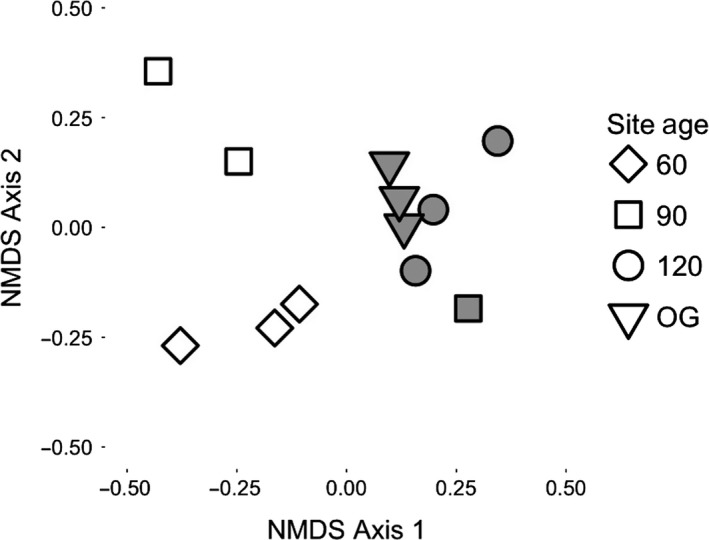
Nonmetric multi‐dimensional scaling for the total dataset using Jaccard abundance index (stress value 0.109). Hollow points are mainland sites and filled points are island sites

Bird assemblages from sites of the same age were more similar to one another, in terms of species composition, than to sites of different ages (Figure 1). One exception was one of the 90‐year‐old sites that was closer in composition to the older sites than to sites of the same age (Figure 1). This is likely a result of the isolation‐related extirpations and shifts in species abundances on BCI, which has caused a strong split between sites along axis 1.

Both forest age and isolation significantly affected bird assemblage composition (PERMANOVA; Table 1), supporting the qualitative patterns shown in the NMDS. There was still a significant effect of isolation on assemblage composition when the analysis was conducted with the IE dataset (i.e., with island‐extirpated species removed; Table 1). This suggests that isolation has impacts beyond the changes in species richness observed at the island sites, with shifts in the relative abundance of remaining species.

**Table 1:** Summary of Permutational Multivariate Analysis of Variance (PERMANOVA) tests for forest age and level of isolation (mainland or island) on the whole dataset, and the canopy and understory dataset separately. Tests undertaken with all data (“All”) and with the island‐extirpated species removed (“Island‐Ex”)

Assemblage	Variable	Dataset	*df*	Sums of squares	*F* value	*R* ^2^	*p* value
Whole	Age	All	3	0.23650	2.273	.46	.01
		Island‐Ex	3	0.21217	2.1513	.45	.01
	Isolation	All	1	0.15366	4.2647	.30	.01
		Island‐Ex	1	0.12953	3.7476	.27	.01
Canopy	Age	All	3	0.24749	2.0928	.44	.03
		Island‐Ex	3	0.22724	2.0255	.43	.01
	Isolation	All	1	0.15002	3.6341	.27	.01
		Island‐Ex	1	0.13190	3.3435	.25	.01
Understory	Age	All	3	0.23217	3.038	.53	.01
		Island‐Ex	3	0.16064	2.622	.50	.01
	Isolation	All	1	0.16793	6.2652	.39	.01
		Island‐Ex	1	0.08456	3.5313	.26	.01

### 3.2 Colonization rates of secondary forest will vary between strata

**Figure 2:**
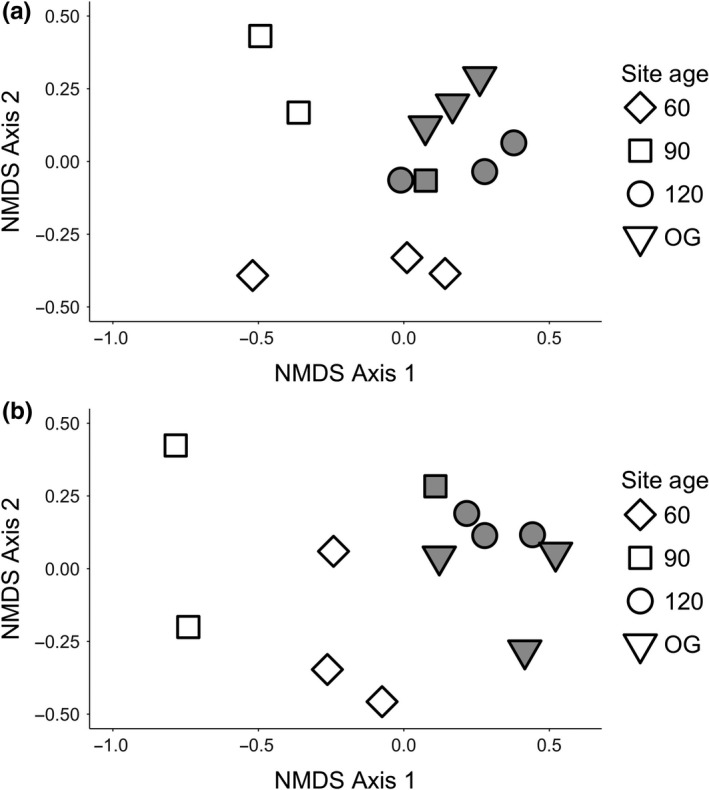
Nonmetric multi‐dimensional scaling using the Jaccard abundance index for (a) canopy assemblages (stress value 0.117), and (b) understory assemblages (stress value 0.088). Hollow points are mainland sites and filled points are island sites.

The utilization‐availability analysis indicated that there were 20 core species in the understory assemblage, and 37 core species in the canopy (Table S1). The canopy assemblages show increased clustering with increasing habitat age, demonstrating greater similarity in species composition over time (Figure 2A; Table 2). The pattern in the understory assemblages is less clear, with no obvious pattern emerging across the chronosequence, suggesting that composition may be influenced by factors other than age (Figure 2B). The differences between the NMDS dissimilarity matrices for the complete dataset and the IE dataset were smaller in the canopy assemblages at the island sites (mean distance 0.015 ± 0.003) compared to the understory assemblages at the island sites (mean distance 0.122 ± 0.016).

**Table 2:** Summed distances between assemblages from the Nonmetric Multi‐dimensional Scaling (NMDS) distance matrices using the Jaccard abundance index

Strata	Forest age			
	60	90	120	Old‐growth
Canopy	1.48	1.38	1.22	0.99
Understory	0.98	1.46	0.97	0.80

Habitat age and isolation impact the composition of both canopy and understory assemblages, with stronger effects observed in the understory assemblage (PERMANOVA; Table 1). Comparing results using the full and IE datasets, there was a considerable reduction in *R* ^2^ for the understory but not the canopy assemblage, suggesting a greater effect of isolation on composition of understory bird assemblages. This finding is likely driven by the proportionally greater number of island‐extirpated species that have been lost from the understory compared to the canopy (5 of 20 species in the understory, compared with 1 of 37 in the canopy).

### 3.3 Colonization rates of secondary forest will vary with feeding guild and body mass

**Figure 3:**
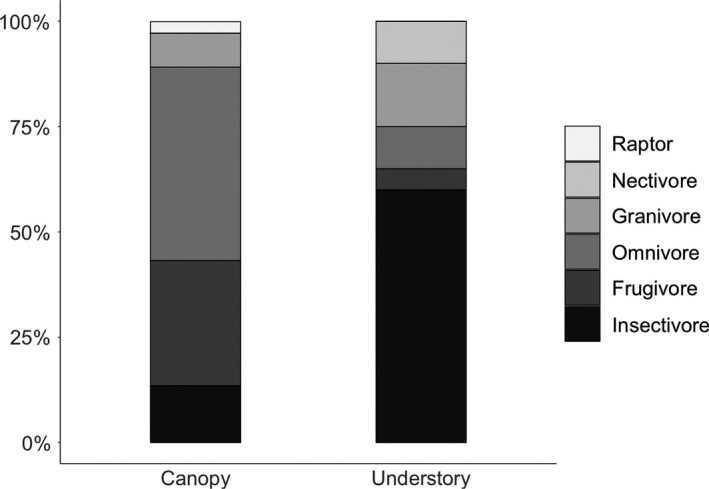
Stacked plot showing guild structure for canopy and understory assemblages. The “age” term was removed from the final models, and so the data are combined across age classes. The canopy is dominated by omnivores, with considerable proportion of frugivores and insectivores; in contrast, the understory assemblage is dominated by insectivores alone

There were significant differences in the guild structure of canopy and understory assemblages (Table S4); this was best explained by guild and strata, although models with forest age were also favored (Table S5). At the level of the individual guilds, forest strata were the best predictor of guild proportions while forest age did not appear to influence composition (Table S5). The guild structure of the canopy assemblage was largely composed of omnivores (46% of species) and frugivores (30% of species); conversely, the understory assemblage was dominated by insectivores (60% of species; Figures 3 and S3).

**Figure 4:**
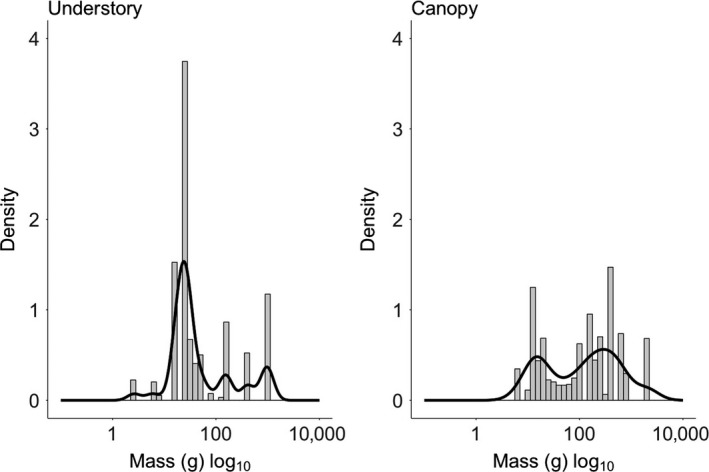
Histograms and kurtosis curves for body mass distributions for understory and canopy strata. (Kurtosis values for understory = 3.03, canopy = 1.84)

Body mass distributions suggested there were differences between strata; the canopy assemblage was composed of birds with a wide range of body masses (platykurtic distribution, 1.84; Figure 4); in contrast, birds in the understory assemblage had more constrained body mass with a distinct peak at 75 g (mesokurtic distribution, 3.03; Figure 4). There were significant differences in mass distribution patterns among the forest ages, strata and body sizes (i.e., small‐ vs. large‐bodied birds) (Tables S6 and S7). These differences were particularly marked when considering body size; mean mass of large‐bodied birds was greater in older forest sites compared to younger forest sites, while this pattern was not evident for small‐bodied birds (Figure S4).

## 4 DISCUSSION

As predicted, both habitat age and isolation influenced avian assemblages at sites in the BCNM; however, it was difficult to disentangle these two effects. The assemblage of birds at any one site was generally more similar to that found at other sites of the same age, compared with assemblages in different aged forest. This suggests that composition is linked to habitat age; however, the picture is strongly influenced by the impact of isolation. Sites isolated on BCI had reduced species diversity and distinct species composition compared to mainland sites, irrespective of forest age. The three 90‐year‐old forest sites illustrate this pattern, where the assemblage at the site isolated on BCI was quite distinct from the two mainland sites. This suggests that successful dispersal into secondary forest is strongly dependent on both suitable mature habitat, and a lack of barriers to colonization (Dunn, 2004; Lees & Peres, 2009).

The extent to which habitat age and isolation effected the composition of bird assemblages varied strongly with forest strata. From our data, the composition of canopy assemblages appears to converge over time irrespective of the level of isolation, suggesting that many canopy species are not inhibited by isolation; this pattern was not seen in understory assemblages. Many Neotropical understory species are known to be highly sensitive to isolation (Lees & Peres, 2008, 2009, 2010; Robinson, 1999, 2001). In the BCNM, isolation has potentially interrupted understory bird community dynamics and resulted in assemblages determined by local extirpations and stochastic processes rather than habitat age.

The guild structures of canopy and understory assemblages were found to differ significantly, with the canopy assemblage dominated by omnivores and the understory dominated by insectivores. However, the proportions of different guilds did not vary with forest age or isolation. Patterns in body mass distribution varied between size classes. Large‐bodied birds were affected by forest age, with greater mean body mass in older forest sites, while small‐bodied birds showed no change with forest age.

### 4.1 Secondary forests as habitats for forest birds; the importance of landscape context

Much of the research examining secondary forest dynamics has focused on very young habitats (<30 years old; Dunn, 2004; Gibson et al., 2011; Gilroy et al., 2014), and numerous studies have emphasized the rapid recovery of many taxa in the first few decades of tropical forest succession (Dunn, 2004). Our study concentrated on older secondary forest than most previous research, enabling us to study development of avian assemblages over a longer timescale. While many bird species are able to colonize secondary forest relatively quickly, there are longer term impacts that should not be underestimated. We found that even after 120 years of forest regeneration, the composition of avian assemblages had not yet converged with those found in old‐growth forest. Our results suggest that despite the rapid changes in vegetation structure during secondary forest development (DeWalt et al., 2003), faunal recovery does not proceed at a comparable rate (Guariguata & Ostertag, 2001; Shankar Raman, Rawat, & Johnsingh, 1998).

Although secondary forest assemblages have not yet converged with those of the old‐growth sites, forest specialists (Stotz et al., 1996) accounted for the majority of species in sites of all ages. This suggests that older secondary forest habitats have high conservation potential. For canopy assemblages, our findings accord with Anderson and Naka (2011), where edge species did not form a significant component of the canopy assemblages in Brazil and Honduras, but contrast with Greenberg (1981) who asserted that a large proportion of the canopy avifauna is comprised of scrubby second‐growth species. However, Greenberg's conclusion may be the result of his method for classifying species’ habitat distribution patterns, which were based on his observations of birds in habitats outside the forest canopy. Given that canopy species tend to be more mobile than understory species, one might be more likely to observe them in secondary growth. Thus, canopy species are not necessarily “scrubby second‐growth” species, indeed most fit our criteria as forest specialists, but they are perhaps more adaptable than species in other strata.

Colonization by canopy species did not appear to be influenced by isolation. Species that forage in the canopy are adapted to cope with strong spatial and temporal variation in resource availability (Winkler & Preleuthner, 2001). In consequence, canopy species can fly considerable distances in order to find food. Many canopy species may visit younger forest as they move across a landscape (Graham, 2001; Neuschulz, Brown, & Farwig, 2013; Walther, 2000) and may be able to cross between BCI and the mainland (minimum distance 200 m; Graham, 2001; Lees & Peres, 2008; Moore et al., 2008; Tscharntke et al., 2008). Thus, while some canopy species might become extirpated from a forest patch, due to environmental changes or insufficient habitat, it is likely that these species are capable of moving among forest patches (Díaz Vélez, Silva, Pizo, & Galetto, 2015; Stouffer et al., 2011). Provided that the requisite resources, such as food or nesting sites, are present in secondary habitat, then canopy species may be able to recolonize. In this way, species diversity in the canopy might be maintained even in fragmented landscapes, as individuals move from patch to patch utilizing the full landscape rather than single habitat fragments. Canopy bird assemblages have been found to exhibit lower genetic differentiation among sites compared to understory assemblages (Burney & Brumfield, 2009), indicating that greater dispersal ability of canopy‐dwelling species maintains gene flow across populations, while understory assemblages remain isolated. Hence, a landscape with small patches of secondary forest may be functionally connected for canopy birds even if physically fragmented (Díaz Vélez et al., 2015), and will have conservation value for canopy species (Neuschulz et al., 2013; Stouffer et al., 2011). Any such functional connectivity will depend on the landscape configuration and matrix quality, and there will likely be a fragmentation threshold for each species beyond which they will struggle to persist (Andrén, 1994; Martensen, Ribeiro, Banks‐Leite, Prado, & Metzger, 2012).

Understory bird assemblages were strongly influenced by isolation in the BCNM. Many understory species are highly dispersal limited, which may drive divergence rather than convergence in assemblage composition over time (Lees & Peres, 2008, 2009, 2010; Robinson, 1999, 2001; Stouffer & Bierregaard, 1995; Tarwater, 2012; Woltmann, Sherry, & Kreiser, 2012). The scale at which landscape composition becomes critical in determining avian colonization will depend on the dispersal abilities of individual species, but generally, understory species are likely to be more affected by the immediate landscape context than canopy species (Stouffer & Bierregaard, 1995, 2007; Wolfe, Stouffer, Mokross, Powell, & Anciaes, 2015). Many understory species are so averse to crossing open habitat that they are unable to colonize secondary forests unless the patch is contiguous with existing mature forest; these species would be incapable of crossing the 200 m between BCI and the mainland (Laurance et al., 2004; Moore et al., 2008). Consequently, understory bird assemblages in secondary forests may be relatively unstable (Stouffer et al., 2011); with highly variable compositions and novel guild structures, which may impact on ecosystem processes (Schleuning et al., 2011; Stouffer & Bierregaard, 1995).

### 4.2 Functional composition of bird assemblages in secondary forest

Despite shifts in species composition, both guild structure and body mass patterns were conserved across the different age classes. The guild structure pattern found in the BCNM matched those reported for other sites in the Neotropics, where omnivores dominated the canopy in Honduras and Brazil (Anderson & Naka, 2011), and insectivores the understory in Costa Rica, Colombia, Panama, Brazil, and French Guiana (Blake & Loiselle, 2001; Castaño‐Villa, Ramos‐Valencia, & Fontúrbel, 2014; Karr, 1990; Modena, Rodrigues, & Souza, 2013; Thiollay, 1994). Understory insectivores are known to be highly sensitive to habitat disturbance and slow to colonize secondary forest (Barlow, Mestre, Gardner, & Peres, 2007), which supports the slower and less predictable reassembly of understory versus canopy bird assemblages in our study.

This study found an influence of body mass on species presence; large‐bodied birds were more numerous in the older sites. This accord with patterns seen in regenerating forest at La Selva, Costa Rica, where those species that exhibited increasing populations over secondary succession had larger mean body mass than those species which declined (Boyle & Sigel, 2015). However, BCNM is strictly protected from poaching, and the older forest sites on BCI are more readily protected than the younger sites on the outlying peninsulas. This protection is likely to strongly influence the populations of the largest birds which inhabit the older sites, particularly Great Tinamou and Crested Guan (Robinson et al., 2000). More generally, outside protected reserves, large‐bodied birds are frequently targeted by hunters (Wright, 2003), and the competing anthropogenic pressures of poaching and protection are possibly of greater importance than forest age in determining the presence of the largest species. This trend for more large‐bodied birds in older forest contrasts with Sigel, Robinson, and Sherry (2010), who reported no predictable influence of body mass on extirpations at either BCI or La Selva. Assuming that secondary forests are adequately protected, and the landscape context is sufficient to permit colonization of all species, irrespective of preferred habitat strata, forests as young as 60 years old may develop avian assemblages with guild structure and body mass patterns similar to those found in old‐growth forest.

## 5 CONCLUSIONS

We have used a novel methodology to demonstrate that avian responses to secondary forest age and isolation vary between canopy and understory bird assemblages. Canopy assemblages were characterized by higher species diversity, and greater variation in dietary niche and body mass than understory species. Thus, canopy birds are likely to be more readily adaptable to suboptimal conditions in secondary habitats than understory species. In addition, many canopy species are able to move across open areas, and so for these species, landscapes may remain functionally connected even if physically fragmented. Secondary forest can therefore play an important role in bird conservation in the Neotropics, particularly for canopy specialists.

## CONFLICT OF INTEREST

The authors declare no conflict of interest.

## AUTHOR CONTRIBUTIONS

TBL and DD conceived and designed the study, TBL and NG collected the data, and TBL and DD analyzed the data. All authors were involved in drafting the article and approved the final version.

## DATA AVAILABILITY

The data collected for this study have been archived at DataSTORRE, the University of Stirling's online repository for research data, available at http://hdl.handle.net/11667/107.
