# Supplementary material for: Canopy bird assemblages are less influenced by habitat age and isolation than understory bird assemblages in Neotropical secondary forest
Source: Ecol Evol. 2018 Apr 27;8(11):5586–97. doi: 10.1002/ece3.4086 (PMC6010736; doi:10.1002/ece3.4086)
Supplement: Supplementary file 1 [file ECE3-8-5586-s001.pdf]

SUPPORTING INFORMATION

TABLE S1. Descriptive statistics summary. “Rarefied species richness” is based on 350 individuals, the smallest number of detections at a single site. “Forest Specialists” are those species categorized by Stotz et al. (1996) as having ‘F1 – Tropical Lowland Evergreen Forest’ as their primary preferred habitat.

**Site**	**Mainland or Island**	**Age**	**Total Number of Species**	**Understory Species**	**Mid-level Species**	**Canopy Species**	**Rarefied species richness (± se)**	**% Abundance of Forest Specialists**	**% Richness of Forest Specialists**	**Simpson Diversity Index**	**Shannon Diversity Index**	**Pielou's Evenness**
**1**	Mainland	60	78	14	42	22	74 ± 1.8	75	79	0.975	3.979	0.91
**2**	Mainland	60	76	11	39	26	73 ± 1.8	72	76	0.974	3.966	0.91
**3**	Mainland	60	79	13	44	22	74 ± 1.8	79	78	0.979	4.062	0.92
**Sub Tot.**			100	15	54	31		75	75			
**4**	Mainland	90	99	15	54	30	91 ± 2.3	80	80	0.985	4.364	0.94
**5**	Mainland	90	91	16	46	29	85 ± 2.1	80	80	0.982	4.231	0.93
**6**	Island	90	88	11	48	29	78 ± 2.5	76	73	0.978	4.101	0.91

**Sub Tot.**			128	19	74	35		78	78			
**7**	Island	120	81	10	46	25	73 ± 2.3	77	78	0.977	4.023	0.91
**8**	Island	120	74	11	39	24	70 ± 1.7	81	80	0.976	3.982	0.92
**9**	Island	120	85	12	45	28	76 ± 2.4	76	79	0.979	4.080	0.91
**Sub Tot.**			109	13	65	31		79	78			
**10**	Island	OG	79	8	43	28	73 ± 2.1	84	84	0.975	3.983	0.90
**11**	Island	OG	75	8	40	27	69 ± 2.0	84	79	0.975	3.953	0.91
**12**	Island	OG	85	11	47	27	77 ± 2.3	82	80	0.980	4.138	0.92
**Sub Tot.**			99	13	57	29		83	83			

TABLE S2. Species list with details of strata membership and feeding guild. Taxonomy follows that of the American Ornithologists' Union checklist (checklist.aou.org). Species with the Common Name marked with an asterisk are "Island Extirpated", i.e., those detected at mainland sites but known to be missing from BCI (Robinson 1999; Willis & Eisenmann 1979). Species marked with a '+' are 'Forest specialists', i.e., those whose primary preferred habitat association is with 'F1 – Tropical Lowland Evergreen Forest' (Stotz et al. 1996), species marked with a '#' are those generally unassociated with this habitat. Species were categorized as inhabiting one of the three strata based on Neu et al.'s (1973) utilization-availability analysis. Those species which showed no apparent preference for a particular stratum were assigned to the 'Middle' level and are designated with an asterisk, those with

fewer than 3 detections could not be tested for a preference and are marked '-'. Presence in each of the four age classes is denoted by an 'x'. Guild assignment followed details in Ridgely & Gwynne (1989) and Angher & Dean (2010), guild codes are 'F' – frugivore, 'G' – granivore, 'I' – insectivore, 'O' – omnivore, 'N' – nectarivore, and 'R' – raptor.

**Family** ***& Species***	**Common Name**	**Stratum**	**60**	**90**	**120**	**OG**	**Guild**
**Tinamidae**							
Tinamus major	Great Tinamou +	Lower	x	x	x	x	G
**Cracidae**							
Penelope purpurascens	Crested Guan +	Upper	x	x	x	x	F
**Accipitridae**							
Leptodon cayanensis	Gray-headed Kite +	-			x		R
Harpagus bidentatus	Double-toothed Kite +	Middle*	x	x	x	x	R
Accipiter bicolor	Bicolored Hawk	-		x			R
Geranospiza caerulescens	Crane Hawk #	-			x		R
Leucopternis semiplumbeus	Semiplumbeous Hawk +	Middle*			x		R
Pseudastur albicollis	White Hawk +	-		x			R
Buteo albonotatus	Zone-tailed Hawk	-			x		R
**Falconidae**							
Micrastur semitorquatus	Collared Forest-falcon +	Middle	x	x	x	x	R
Falco rufigularis	Bat Falcon +	-		x		x	R
**Rallidae**							
Aramides cajaneus	Gray-necked Wood-rail #	Lower	x	x	x	x	O
**Columbidae**							
Patagioenas nigrirostris	Short-billed Pigeon +	Upper	x	x		x	F
Patagioenas cayennensis	Pale-vented Pigeon	Upper	x	x	x	x	F
Patagioenas speciosa	Scaled Pigeon +	Upper	x	x	x	x	F
Leptotila cassinii	Gray-chested Dove +	Lower	x	x	x	x	F

Geotrygon montana	Ruddy Quail-dove +	Lower	x			x	F
**Psittacidae**								
Pionus menstruus	Blue-headed Parrot	Upper	x	x	x	x	G
Amazona autumnalis	Red-lored Parrot +	Upper	x	x	x	x	G
Amazona farinosa	Mealy Parrot +	Upper	x	x	x	x	G
**Cuculidae**								
Piaya cayana	Squirrel Cuckoo +	Middle	x	x	x	x	I
Dromococcyx phasianellus	Pheasant Cuckoo +	Lower	x	x		x	I
**Strigidae**								
Pulsatrix perspicillata	Spectacled Owl	Upper	x	x	x	x	R
Ciccaba virgata	Mottled Owl +	Middle	x	x	x	x	R
Megascops guatemalae	Vermiculated Screech-owl +	Middle*		x	x		R
**Nyctibiidae**								
Nyctibius grandis	Great Potoo +	Middle		x	x	x	I
Nyctibius griseus	Common Potoo +	Middle*	x	x		x	I
**Trochilidae**								
Phaethornis longirostris	Long-billed Hermit +	Lower	x	x	x	x	N
Phaethornis striigularis	Stripe-throated Hermit +	Lower	x	x	x		N
Florisuga mellivora	White-necked Jacobin +	Middle*		x	x	x	N
Anthracothorax nigricollis	Black-throated Mango*	-		x			N
Juliamyia julie	Violet-bellied Hummingbird +	Middle	x	x	x	x	N
Lepidopyga coeruleogularis	Sapphire-throated Hummingbird +	-				x	N
Amazilia amabilis	Blue-chested Hummingbird +	Middle	x	x	x	x	N
Heliothryx barroti	Purple-crowned Fairy +	Middle*	x	x	x	x	N
**Trogonidae**								
Trogon chionurus	White-tailed Trogon +	Middle*		x	x	x	O
Trogon caligatus	Gartered Trogon +	Middle	x	x	x	x	O
Trogon melanrus	Black-tailed Trogon +	Middle*	x	x	x	x	O
Trogon rufus	Black-throated Trogon +	Middle	x	x	x	x	O

Trogon massena	Slaty-tailed Trogon +	Middle	x	x	x	x	O
**Motmotidae**							
Baryphthengus martii	Rufous Motmot +	Middle	x	x	x	x	O
Electron platyrhynchum	Broad-billed Motmot +	Middle*	x	x	x	x	O
Momotus subrufescens	Whooping Motmot +	Middle	x	x	x		O
**Bucconidae**							
Notharchus pectoralis	Black-breasted Puffbird +	Middle	x	x	x	x	I
Malacoptila panamensis	White-whiskered Puffbird +	Lower	x	x	x	x	I
**Galbulidae**							
Jacamerops aureus	Great Jacamar* +	Middle*		x			I
**Ramphastidae**							
Pteroglossus torquatus	Collared Aracari +	Upper	x			x	F
Ramphastos sulfuratus	Keel-billed Toucan +	Upper	x	x	x	x	F
Ramphastos swainsonii	Chestnut-mandibled Toucan +	Upper	x	x	x	x	F
**Picidae**							
Melanerpes pucherani	Black-cheeked Woodpecker +	Middle*	x	x		x	O
Celeus loricatus	Cinnamon Woodpecker +	Middle*		x			O
Dryocopus lineatus	Lineated Woodpecker	Middle	x	x	x	x	I
Campephilus melanoleucos	Crimson-crested Woodpecker	Middle	x	x	x	x	I
**Furnariidae**							
Automolus ochrolaemus	Buff-throated Foliage-gleaner +	-			x		I
Xenops minutus	Plain Xenops +	Middle*	x	x	x		I
Sclerurus mexicanus	Tawny-throated Leaftosser +	-		x		x	I
Sclerurus guatemalensis	Scaly-throated Leaftosser +	Lower		x	x	x	I
Dendrocincla fuliginosa	Plain-brown Woodcreeper +	Middle*	x				I
Dendrocincla homochroa	Ruddy Woodcreeper +	Middle*	x	x	x	x	I
Dendrocolaptes sanctithomae	Northern Barred Woodcreeper +	Middle	x	x	x	x	I

Xiphorhynchus susurrans	Cocoa Woodcreeper +	Middle	x	x	x	x	I
Xiphorhynchus lachrymosus	Black-striped Woodcreeper +	Middle	x	x	x	x	I
Glyphorynchus spirurus	Wedge-billed Woodcreeper +	Middle*			x	x	I
Lepidocolaptes souleyetii	Streak-headed Woodcreeper	Middle*	x	x	x		I
**Thamnophilidae**							
Thamnophilus atrinucha	Black-crowned Antshrike	Lower	x	x	x	x	I
Dysithamnus puncticeps	Spot-crowned Antvireo +	-			x		I
Epinecrophylla fulviventris	Checker-throated Antwren +	Middle*	x	x	x	x	I
Myrmotherula axillaris	White-flanked Antwren +	Middle	x	x	x	x	I
Microrhopias quixensis	Dot-winged Antwren +	Middle	x	x	x	x	I
Cercomacroides tyrannina	Dusky Antbird +	-		x			I
Myrmeciza exsul	Chestnut-backed Antbird +	Lower	x	x	x	x	I
Hylophylax naevioides	Spotted Antbird +	Lower	x	x	x	x	I
Gymnopithys bicolor	Bicolored Antbird +	Lower	x	x	x	x	I
Phaenostictus mcleannani	Ocellated Antbird* +	Lower		x			I
**Formicariidae**							
Formicarius analis	Black-faced Antthrush* +	Lower	x	x			I
**Tyrannidae**							
Ornithion brunneicapillus	Brown-capped Tyrannulet +	Middle	x	x	x	x	O
Camptostoma obsoletum	Southern Beardless-tyrannulet #	Middle*	x	x	x	x	I
Tyrannulus elatus	Yellow-crowned Tyrannulet	Middle	x	x	x	x	I
Myiopagis gaimardii	Forest Elaenia +	Middle*	x	x	x	x	I
Mionectes oleagineus	Ochre-bellied Flycatcher +	Middle*	x			x	O
Zimmerius vilissimus	Paltry Tyrannulet	Middle	x	x	x	x	O
Myiornis atricapillus	Black-capped Pygmy-tyrant +	Middle*		x	x		I

Rhynchocyclus olivaceus	Olivaceous Flatbill +	Middle*	x	x	x	x	I
Tolmomyias assimilis	Yellow-margined Flycatcher +	Middle	x	x	x	x	I
Platyrinchus coronatus	Golden-crowned Spadebill +	-			x		I
Terenotriccus erythrurus	Ruddy-tailed Flycatcher +	Middle*	x	x	x	x	I
Contopus virens	Eastern Wood-pewee +	Middle*	x	x	x	x	I
Empidonax virescens	Acadian Flycatcher +	Middle*	x	x	x	x	I
Attila spadiceus	Bright-rumped Attila +	Middle	x	x	x	x	O
Rhytipterna holerythra	Rufous Mourner +	Middle	x	x	x	x	O
Myiarchus tuberculifer	Dusky-capped Flycatcher	Upper	x	x	x	x	I
Myiarchus panamensis	Panama Flycatcher #	-				x	O
Myiarchus crinitus	Great Crested Flycatcher +	Upper	x	x	x	x	O
Pitangus sulphuratus	Great Kiskadee #	Upper	x	x	x		O
Myiozetetes cayanensis	Rusty-margined Flycatcher	-		x	x		O
Myiozetetes similis	Social Flycatcher +	Upper	x	x	x		O
Myiodynastes maculatus	Streaked Flycatcher +	Middle*	x	x		x	O
Legatus leucophaius	Piratic Flycatcher +	-		x	x		F
Tyrannus melancholicus	Tropical Kingbird	Upper	x	x	x		I
Tyrannus tyrannus	Eastern Kingbird	Upper	x	x			O
Oncostoma olivaceum	Southern Bentbill +	Middle	x	x	x	x	O
**Incertae *sedis***							
Tityra semifasciata	Masked Tityra +	Upper	x	x	x	x	O
**Cotingidae**							
Cotinga nattererii	Blue Cotinga +	Upper		x	x	x	F
Querula purpurata	Purple-throated Fruitcrow +	Upper	x	x	x	x	F
**Pipridae**							
Lepidothrix coronata	Blue-crowned Manakin* +	Lower		x			F

Ceratopipra mentalis	Red-capped Manakin +	Middle	x	x	x	x	F
**Vireonidae**							
Vireo olivaceus	Red-eyed Vireo +	Upper		x	x	x	O
Vireo flavoviridis	Yellow-green Vireo	Middle*		x	x		O
Tunchiornis ochraceiceps	Tawny-crowned Greenlet +	-				x	I
Pachysylvia decurtata	Lesser Greenlet +	Middle	x	x	x	x	O
Vireolanius pulchellus	Green Shrike-vireo* +	Upper	x	x			O
**Troglodytidae**							
Thryophilus rufalbus	Rufous-and-white Wren* +	Lower		x			I
Cyphorhinus phaeocephalus	Song Wren* +	Lower		x			I
**Sylviidae**							
Ramphocaenus melanurus	Long-billed Gnatwren +	Middle*	x	x	x		I
Polioptila plumbea	Tropical Gnatcatcher	Upper	x	x	x	x	I
**Turdidae**							
Catharus ustulatus	Swainson's Thrush	-		x			O
**Parulidae**							
Oreothlypis peregrina	Tennessee Warbler	Upper	x	x	x	x	I
Setophaga pensylvanica	Chestnut-sided Warbler +	Upper		x	x	x	O
Setophaga magnolia	Magnolia Warbler +	-	x	x			O
Setophaga fusca	Blackburnian Warbler #	Middle*		x	x		O
Setophaga castanea	Bay-breasted Warbler +	Middle*		x	x	x	O
Setophaga ruticilla	American Redstart +	Middle*		x			I
Cardellina canadensis	Canada Warbler	Middle*	x	x		x	I
**Thraupidae**							
Eucometis penicillata	Gray-headed Tanager +	Lower	x	x	x	x	I
Tachyphonus luctuosus	White-shouldered Tanager +	Upper	x	x	x	x	O
Thraupis episcopus	Blue-gray Tanager +	-		x			F

Tangara inornata	Plain-colored Tanager +	Upper		x	x	x	O
Tangara larvata	Golden-hooded Tanager* +	-		x			O
Dacnis cayana	Blue Dacnis +	Upper	x	x	x	x	I
Chlorophanes spiza	Green Honeycreeper +	Upper	x	x	x	x	O
Cyanerpes lucidus	Shining Honeycreeper +	Upper		x	x	x	O
Cyanerpes cyaneus	Red-legged Honeycreeper +	Upper	x	x	x	x	O
**Incertae *sedis***							
Saltator grossus	Slate-colored Grosbeak* +	-		x			F
**Cardinalidae**							
Piranga rubra	Summer Tanager #	Middle*	x	x	x	x	O
Piranga olivacea	Scarlet Tanager +	Upper	x	x	x	x	O
Habia fuscicauda	Red-throated Ant-tanager +	Lower	x	x	x		O
Cyanocompsa cyanoides	Blue-black Grosbeak +	Middle*	x	x	x	x	O
**Icteridae**							
Icterus chrysater	Yellow-backed Oriole	Upper	x	x	x	x	O
Icterus galbula	Baltimore Oriole #	Middle*	x	x	x		O
Cacicus uropygialis	Scarlet-rumped Cacique* +	Middle*	x	x			O
Cacicus cela	Yellow-rumped Cacique	Upper	x	x	x	x	O
Psarocolius decumanus	Crested Oropendola* +	-	x				O
Psarocolius wagleri	Chestnut-headed Oropendola +	Upper	x	x	x	x	O
**Fringillidae**							
Euphonia luteicapilla	Yellow-crowned Euphonia +	Upper	x				F
Euphonia fulvicrissa	Fulvous-vented Euphonia +	Upper		x		x	F

TABLE S3. Model selection for distance sampling contrasting both Conventional Distance Sampling (CDS) and Multiple-Covariate Distance Sampling (MCDS) models. Models are sorted by differences in AIC (Buckland et al. 2015; Marques et al. 2007). The simpler CDS models without covariates were preferred over the MCDS models, suggesting that the covariates 'Forest Age' and 'Site' did not influence detectability.

Model	Adjustment terms	Covariate	AIC	ΔAIC
CDS Hazard-rate	cosine	-	21784.08	0
CDS Half-normal	cosine	-	21792.37	8.28
MCDS Hazard-rate	-	Forest Age	21864.80	80.72
Hazard-rate	polynomial	-	21876.83	92.75
Hazard-rate	none	-	21886.43	102.35
MCDS Hazard-rate	-	Site	21888.33	104.25
Uniform	cosine	-	21953.48	169.40
MCDS Half-normal	-	Forest Age	22573.12	789.04
Half-normal	hermite	-	22621.87	837.79
Half-normal	none	-	22628.37	844.28
MCDS Half-normal	-	Site	22629.89	845.80
Uniform	polynomial	-	27129.14	5345.05

TABLE S4. Results of the Generalized Linear Models (GLMs) with a binomial error structure and a logit link for guild proportions. Using all data from core Canopy and Understory assemblages, with the predictors ‘Guild’ (six categories; Frugivore, Granivore, Insectivore, Nectarivore, Omnivore and Raptor), ‘Forest Age’ (four categories; 60-, 90-, 120-years-old, and old-growth), ‘Strata’ (two categories; understory and canopy), and all possible interactions. Followed by further results for each guild tested individually, with ‘Forest Age’, ‘Strata’ and the ‘Forest Age by Strata’ interaction.

	Estimate	Std. error	z value
(Intercept)	-1.88707	0.4799	-3.932
GuildGranivore	-0.56967	0.76955	-0.74
GuildInsectivore	1.99243	0.57953	3.438
GuildNectarivore	0.21309	0.65438	0.326
GuildOmnivore	-0.253	0.71394	-0.354
GuildRaptor	-20.43974	6936.90529	-0.003
Age90	-0.36422	0.71177	-0.512
Age120	-0.41552	0.77264	-0.538
AgeOG	0.13787	0.72373	0.19
StrataCanopy	0.8262	0.55235	1.496
GuildGranivore:Age90	0.25601	1.10793	0.231
GuildInsectivore:Age90	0.64452	0.84321	0.764
GuildNectarivore:Age90	-0.21309	0.99038	-0.215
GuildOmnivore:Age90	0.71253	0.99019	0.72
GuildRaptor:Age90	0.2783	9775.70629	0
GuildGranivore:Age120	0.56967	1.15132	0.495
GuildInsectivore:Age120	0.86977	0.91295	0.953
GuildNectarivore:Age120	-0.21309	1.07775	-0.198
GuildOmnivore:Age120	0.253	1.11492	0.227
GuildRaptor:Age120	0.54092	9848.92917	0
GuildGranivore:AgeOG	0.23942	1.1228	0.213
GuildInsectivore:AgeOG	0.2874	0.88778	0.324

GuildNectarivore:AgeOG	-1.72199	1.32671	-1.298
GuildOmnivore:AgeOG	-0.52353	1.15896	-0.452
GuildRaptor:AgeOG	0.16932	9896.13054	0
GuildGranivore:StrataCanopy	-0.28311	0.89135	-0.318
GuildInsectivore:StrataCanopy	-2.50709	0.715	-3.506
GuildNectarivore:StrataCanopy	-22.04684	6789.00412	-0.003
GuildOmnivore:StrataCanopy	0.9084	0.80251	1.132
GuildRaptor:StrataCanopy	18.39453	6936.90532	0.003
Age90:StrataCanopy	0.32648	0.80126	0.407
Age120:StrataCanopy	0.36038	0.86118	0.418
AgeOG:StrataCanopy	0.09883	0.81001	0.122
GuildGranivore:Age90:StrataCanopy	-0.47684	1.2705	-0.375
GuildInsectivore:Age90:StrataCanopy	-0.78379	1.01844	-0.77
GuildNectarivore:Age90:StrataCanopy	18.67954	6789.00424	0.003
GuildOmnivore:Age90:StrataCanopy	-0.4059	1.10501	-0.367
GuildRaptor:Age90:StrataCanopy	-0.47852	9775.70633	0
GuildGranivore:Age120:StrataCanopy	-0.62317	1.31283	-0.475
GuildInsectivore:Age120:StrataCanopy	-0.83304	1.08225	-0.77
GuildNectarivore:Age120:StrataCanopy	18.83211	6789.00425	0.003
GuildOmnivore:Age120:StrataCanopy	-0.08008	1.22485	-0.065
GuildRaptor:Age120:StrataCanopy	-0.58516	9848.92921	0
GuildGranivore:AgeOG:StrataCanopy	-0.65571	1.2827	-0.511
GuildInsectivore:AgeOG:StrataCanopy	-0.81334	1.06116	-0.766

GuildNectivore:AgeOG:StrataCanopy	19.98546	6789.0043	0.003
GuildOmnivore:AgeOG:StrataCanopy	0.34746	1.25908	0.276
GuildRaptor:AgeOG:StrataCanopy	-0.57077	9896.13058	0

**Guild**		**Estimate**	**Std. Error**	**z value**
**Frugivores**	Intercept	-1.88707	0.47990	-3.932
	Age90	-0.36422	0.71177	-0.512
	Age120	-0.41552	0.77264	-0.538
	AgeOG	0.13787	0.72373	0.190
	StrataCanopy	0.82629	0.55235	1.496
	Age90:StrataCanopy	0.32648	0.80126	0.407
	Age120:StrataCanopy	0.36038	0.86118	0.418
	AgeOG:StrataCanopy	0.09883	0.81001	0.122
**Granivores**	Intercept	-2.4567	0.6016	-4.084
	Age90	-0.1082	0.8490	-0.127
	Age120	0.1542	0.8536	0.181
	AgeOG	0.3773	0.8584	0.440
	StrataCanopy	0.5431	0.6996	0.776
	Age90:StrataCanopy	-0.1504	0.9860	-0.152
	Age120:StrataCanopy	-0.2628	0.9909	-0.265
	AgeOG:StrataCanopy	-0.5569	0.9946	-0.560

**Insectivores**	Intercept	0.1054	0.3249	0.324
	Age90	0.2803	0.4521	0.620
	Age120	0.4543	0.4863	0.934
	AgeOG	0.4253	0.5142	0.827
	StrataCanopy	-1.6809	0.4540	-3.702
	Age90:StrataCanopy	-0.4573	0.6287	-0.727
	Age120:StrataCanopy	-0.4727	0.6555	-0.721
	AgeOG:StrataCanopy	-0.7145	0.6855	-1.042
**Omnivores**	Intercept	-2.14007	0.52859	-4.049
	Age90	0.34831	0.68837	0.506
	Age120	-0.16252	0.80378	-0.202
	AgeOG	-0.38566	0.90520	-0.426
	StrataCanopy	1.73460	0.58218	2.979
	Age90:StrataCanopy	-0.07942	0.76095	-0.104
	Age120:StrataCanopy	0.28030	0.87099	0.322
	AgeOG:StrataCanopy	0.44629	0.96393	0.463
**Nectarivores**	Intercept	-1.6740	0.449	-3.763
	Age90	-0.5773	0.6886	-0.838
	Age120	-0.6286	0.7514	-0.837
	AgeOG	-1.5841	1.1119	1.425
	StrataCanopy	-20.2206	4117.7392	-0.005
	Age90:StrataCanopy	18.0060	4117.7393	0.004

	Age120:StrataCanopy	18.1925	4117.7393	0.004
	AgeOG:StrataCanopy	19.0843	4117.7394	0.005
**Raptors**	Intercept	-27.33	84510.00	0
	Age90	-0.09	119100.00	0
	Age120	0.13	120000.00	0
	AgeOG	0.31	120600.00	0
	StrataCanopy	24.22	84510.00	0
	Age90:StrataCanopy	-0.15	119100.00	0
	Age120:StrataCanopy	-0.22	120000.00	0
	AgeOG:StrataCanopy	-0.47	120600.00	0

TABLE S5. Results of the GLM selection for feeding guilds, with models ranked by AICc. An 'X' indicates that the predictor was included in the model. First, model selection for the core canopy and understory assemblages combined, and then each guild individually.

**Model**	**Age**	**Guild**	**Strata**	**Age:Guild**	**Age:Strata**	**Guild:Strata**	**Age:Guild:Strata**	**df**	**logLik**	**AICc**	**Δ AICc**	w_i
39		X	X			X		12	-173.388	373.2	0	0.975
40	X	X	X			X		15	-173.384	380.5	7.36	0.025
56	X	X	X		X	X		18	-173.382	388.2	15.08	0.001
48	X	X	X	X		X		30	-170.943	418.3	45.19	0
64	X	X	X	X	X	X		33	-170.874	428.1	54.99	0
128	X	X	X	X	X	X	X	48	-168.334	482.2	109.03	0
3		X						6	-264.854	542.3	169.16	0
7		X	X					7	-264.848	544.5	171.36	0
4	X	X						9	-264.85	549	175.89	0
8	X	X	X					10	-264.845	551.3	178.19	0
24	X	X	X		X			13	-264.843	558.5	185.33	0
12	X	X		X				24	-261.628	581.3	208.18	0
16	X	X	X	X				25	-261.623	584.3	211.1	0
32	X	X	X	X	X			28	-261.621	593.4	220.21	0
1								1	-418.63	839.3	466.13	0
5			X					2	-418.625	841.3	468.18	0
2	X							4	-418.627	845.5	472.38	0
6	X		X					5	-418.622	847.7	474.52	0
22	X		X		X			8	-418.62	854.3	481.15	0

	Model	Age	Strata	Age:Strata	df	logLik	AICc	ΔAICc	w_i
**Frugivores**	3		X		2	-36.454	77.5	0.00	0.973
	4	X	X		5	-35.762	84.9	7.38	0.024
	1				1	-43.521	89.2	11.74	0.003
	2	X			4	-42.577	95.3	17.78	0.000
	8	X	X	X	8	-35.630	96.9	19.38	0.000
**Granivores**	1				1	-29.568	61.3	0.00	0.672
	3		X		2	-29.153	62.9	1.56	0.308
	2	X			4	-29.407	68.9	7.60	0.015
	4	X	X		5	-28.999	71.3	10.01	0.004
	8	X	X	X	8	-28.833	83.3	21.95	0.000
**Insectivores**	3		X		2	-38.137	80.8	0.00	0.984
	4	X	X		5	-37.892	89.1	8.27	0.016
	8	X	X	X	8	-37.301	100.2	19.36	0.000
	1				1	-81.744	165.7	84.82	0.000
	2	X			4	-81.340	172.8	91.94	0.000
**Nectarivores**	3		X		2	-20.547	45.7	0.00	0.976
	4	X	X		5	-19.883	53.1	7.43	0.024
	8	X	X	X	8	-18.391	62.4	16.72	0.000
	1				1	-30.703	63.6	17.92	0.000
	2	X			4	-29.595	69.3	23.63	0.000

**Omnivores**	3		X		2	-37.268	79.1	0.00	0.977
	4	X	X		5	-36.622	86.6	7.47	0.023
	8	X	X	X	8	-63.029	98.4	19.28	0.000
	1				1	-63.029	128.2	49.13	0.000
	2	X			4	-62.457	135.0	55.91	0.000
**Raptors**	3		X		2	-11.829	28.2	0.00	0.951
	1				1	-16.289	34.8	6.53	0.036
	4	X	X		5	-11.785	36.9	8.67	0.012
	2	X			4	-16.252	42.6	14.38	0.001
	8	X	X	X	8	-11.785	49.2	20.94	0.000

TABLE S6. Results of the Generalized Linear Models (GLMs) with a poisson error structure and a log link for body mass distributions. Using all data from core Canopy and Understory assemblages, with the predictors ‘Guild’ (six categories; Frugivore, Granivore, Insectivore, Nectarivore, Omnivore and Raptor), ‘Forest Age’ (four categories; 60-, 90-, 120-years-old, and old-growth), ‘Strata’ (two categories; understory and canopy), and all possible interactions.

	**Estimate**	**Std. Error**	**t value**
(Intercept)	2735.3	2790.64	0.98
Age90	33.12	3890.79	0.009
Age120	-387.99	4125.89	-0.094
AgeOG	-327.18	4118.76	-0.079
StrataCanopy	455.23	3716.95	0.122
SizeLarge	47780.2	5299	9.017
Age90:StrataCanopy	-60.32	5022.42	-0.012
Age120:StrataCanopy	-382.29	5341.26	-0.072
AgeOG:StrataCanopy	-98.9	5356	-0.018
Age90:SizeLarge	5429.16	7635.34	0.711
Age120:SizeLarge	12507.6	8244.29	1.517
AgeOG:SizeLarge	12812.49	7777.74	1.647
StrataCanopy:SizeLarge	-7717.95	6228.99	-1.239
Age90:StrataCanopy:SizeLarge	2494.18	8773.95	0.284
Age120:StrataCanopy:SizeLarge	7920.91	9390.44	0.844
AgeOG:StrataCanopy:SizeLarge	6971.85	8964.58	0.778

TABLE S7. Results of the GLM selection for body mass distribution, with models ranked by AICc. An 'X' indicates that the predictor was included in the model.

**Model**	**Age**	**BodySize**	**Strata**	**Age:BodySize**	**Age:Strata**	**BodySize:Strata**	**Age:BodySize:Strata**	**df**	**logLik**	**AICc**	**Δ AICc**	w_i

12	X	X		X				9	-41852.53	83723.1	0	0.562
16	X	X	X	X				10	-41852.37	83724.8	1.68	0.243
48	X	X	X	X		X		11	-41851.72	83725.5	2.4	0.169
32	X	X	X	X	X			13	-41852.15	83730.4	7.29	0.015
64	X	X	X	X	X	X		14	-41851.51	83731.1	8.03	0.01
128	X	X	X	X	X	X	X	17	-41851.01	83736.2	13.08	0.001
4	X	X						6	-41870.73	83753.5	30.37	0
8	X	X	X					7	-41870.6	83755.2	32.11	0
24	X	X	X		X			10	-41867.95	83756	32.84	0
40	X	X	X			X		8	-41870.28	83756.6	33.48	0
56	X	X	X		X	X		11	-41867.62	83757.3	34.21	0
3		X						3	-41886	83778	54.88	0
7		X	X					4	-41885.97	83779.9	56.83	0
39		X	X			X		5	-41885.76	83781.5	58.41	0
22	X		X		X			9	-42510.7	85039.5	1316	0
6	X		X					6	-42514.76	85041.5	1318	0
5			X					3	-42533.66	85073.3	1350	0
2	X							5	-42553.28	85116.6	1393	0
1								2	-42574.62	85153.2	1430	0

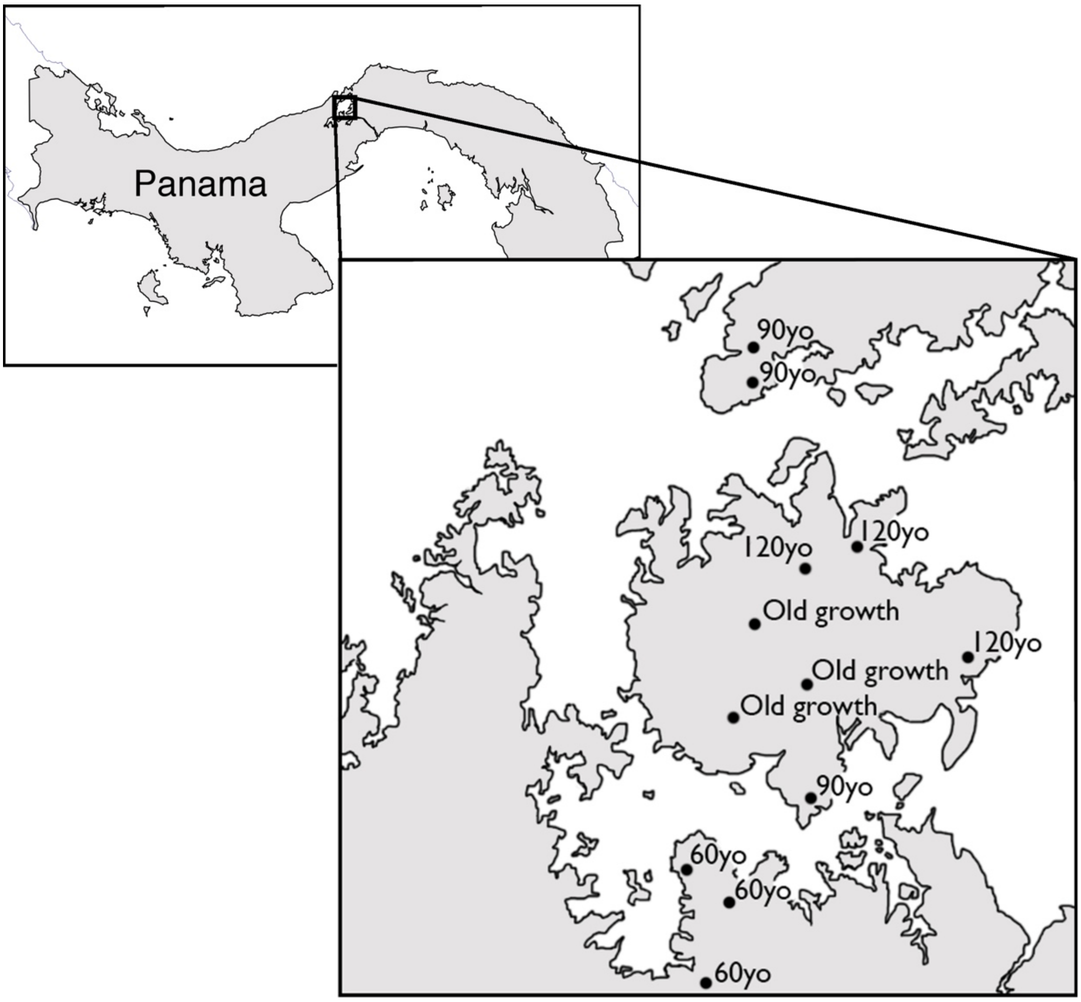

FIGURE S1. Map of the study area

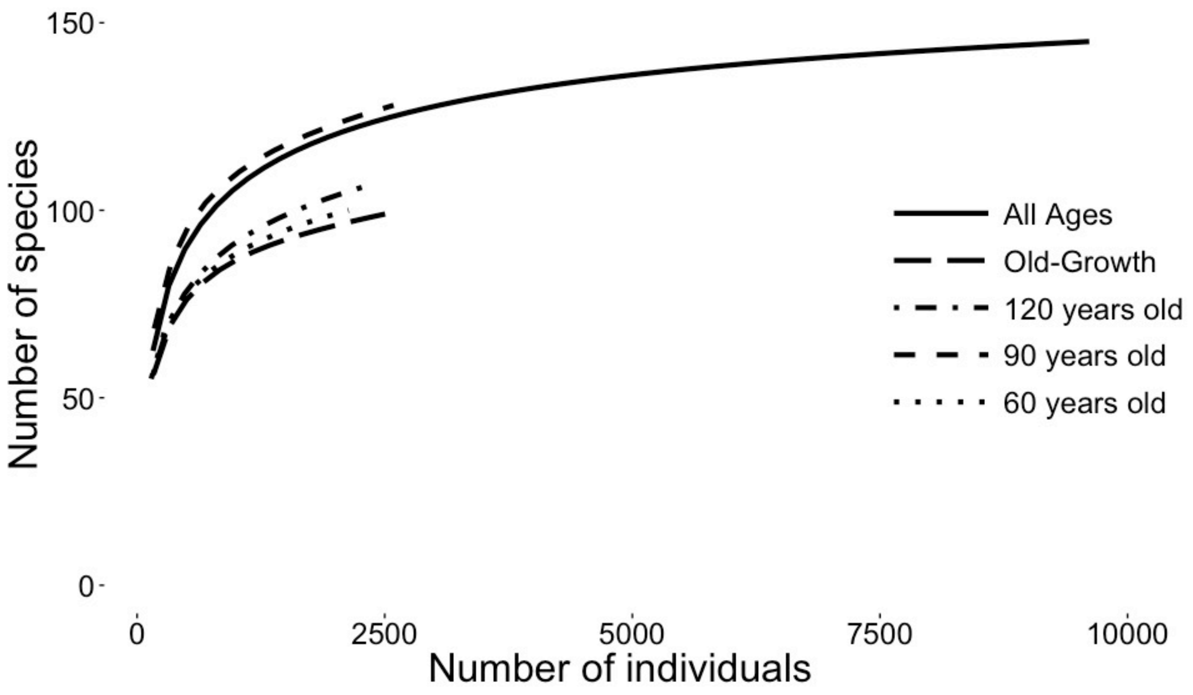

FIGURE S2. Species rarefaction curves for the total landscape and each age class separately. Rarefaction was based on 350 individuals, the minimum number detected at a single site. All curves for the separate age classes are beginning to level off, suggesting that the majority of species had been detected at each individual site, and that only some rare species were yet to be detected. The curve for all sites combined has reached the asymptote, implying that at the landscape scale, surveys were sufficient to characterize the bird community.

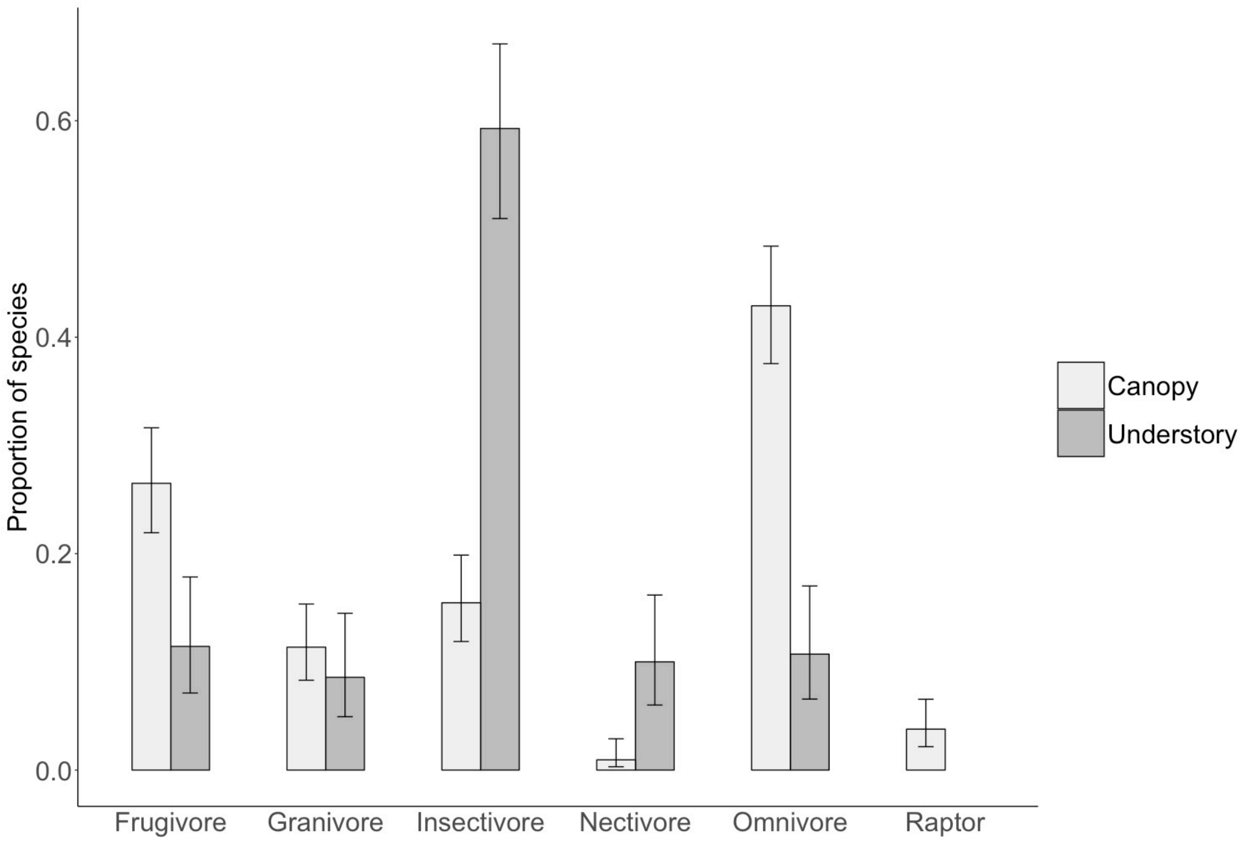

FIGURE S3. Predicted values with 95% confidence intervals from the GLM showing differences in guild proportions between canopy and understory assemblages.

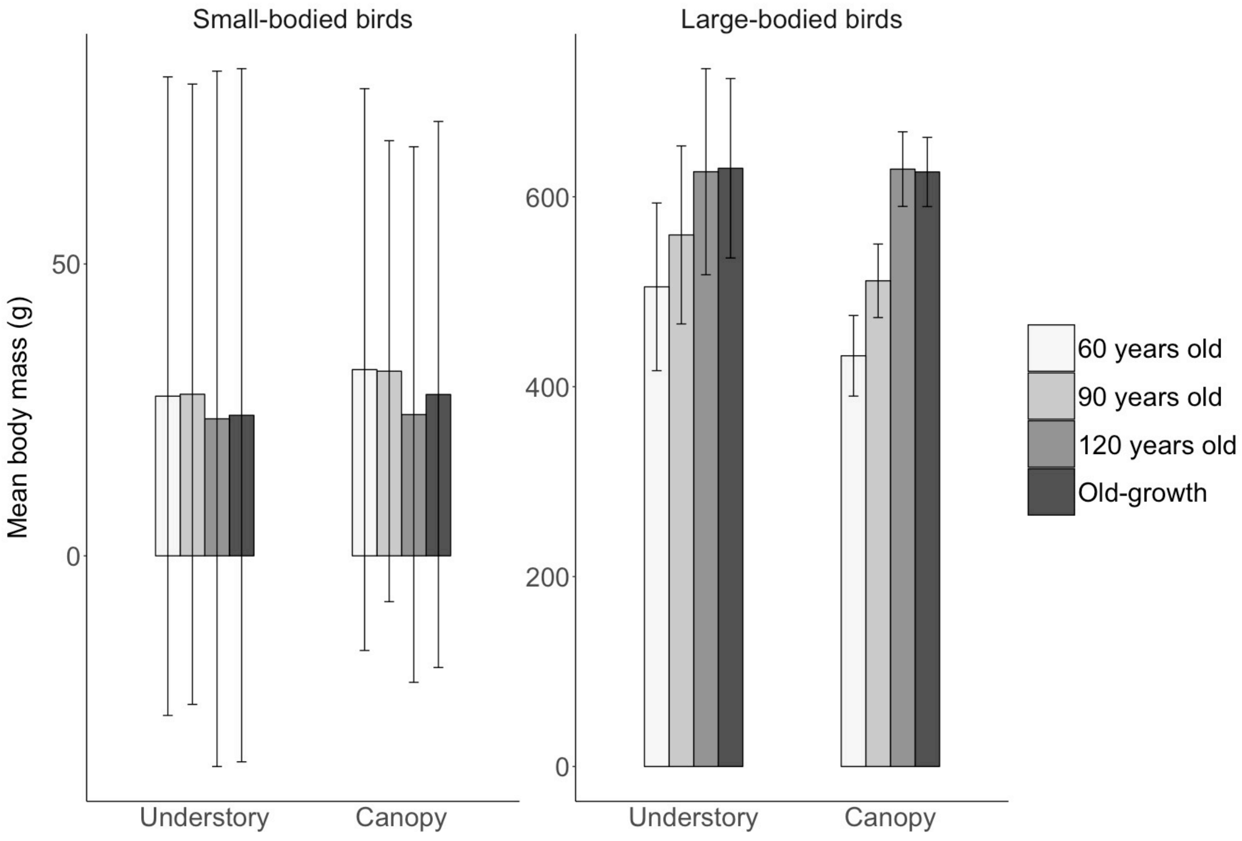

FIGURE S4. Predicted values with 95% confidence intervals from the GLM, showing body mass distributions between strata and among age classes for small- and large-bodied birds.
